# Augmented reality as a novel approach for addiction treatment: development of a smoking cessation app

**DOI:** 10.1080/07853890.2022.2140451

**Published:** 2022-11-08

**Authors:** Min-Jeong Yang, Karen O. Brandon, Steven K. Sutton, Marloes Kleinjan, Leslie E. Sawyer, Thomas H. Brandon, Christine Vinci

**Affiliations:** a Department of Health Outcomes and Behavior, Moffitt Cancer Center, Tampa, FL, USA; b Department of Psychology, University of South Florida, Tampa, FL, USA; c Department of Oncologic Sciences, University of South Florida, Tampa, FL, USA; d Department of Biostatistics and Bioinformatics, Moffitt Cancer Center, Tampa, FL, USA; e Department of Child and Adolescent Health, Trimbos Institute, Utrecht, The Netherlands; f Department of Interdisciplinary Social Science, Utrecht University, Utrecht, The Netherlands

**Keywords:** Cue exposure therapy, smoking, smartphone app, urge, augmented reality, addiction, extinction

## Abstract

**Objective:**

Augmented reality (AR) is a rapidly developing technology that has substantial potential as a novel approach for addiction treatment, including tobacco use. AR can facilitate the delivery of cue exposure therapy (CET) such that individuals can experience the treatment in their natural environments as viewed via a smartphone screen, addressing the limited generalizbility of extinction learning. Previously, our team developed a basic AR app for smoking cessation and demonstrated the necessary mechanisms for CET. Specifically, we showed that the AR smoking cues, compared to neutral cues, elicited substantial cue reactivity (i.e. increased urge) and that repeated exposure to the AR smoking cues reduced urge (i.e. extinction) in a laboratory setting. Here we report the next step in the systematic development of the AR app, in which we assessed the usability and acceptability of the app among daily smokers in their natural environments.

**Method:**

Daily smokers (*N* = 23, 78.3% female, Mean Age = 43.4, Mean Cigarettes/Day = 14.9), not actively quitting, were instructed to use the AR app in locations and situations where they smoke (e.g. home, bar) at least 5 times per day over one week. The study is registered in clinicaltrials.gov (NCT04101422).

**Results:**

Results indicated high usability and acceptability. Most of the participants (73.9%) used the AR app on at least 5 days. Participants found the AR cues realistic and well-integrated in their natural environments. The AR app was perceived as easy to use (Mean = 4.1/5) and learn (mean of 2 days to learn). Overall satisfaction with the app was also high. Secondary analyses found that 56.5% reported reduced smoking, with an average 26% reduction in cigarettes per day at follow-up.

**Conclusions:**

These findings set the stage for a randomized controlled trial testing the AR app as an adjuvant therapy for treating tobacco dependence, with potential applicability to other substances.

**KEY MESSAGE**
- This study found that the augmented reality (AR) smartphone application that utlized cue exposure treatment for smoking cessation was perceived as easy to use and learn in the natural, day-to-day environment of daily smokers. Findings set the stage for a larger clinical trial testing the AR app as an adjuvant therapy for treating tobacco dependence, with potential applicability to other addictive behaviors.

## Introduction

Cigarette smoking remains the leading cause of cancer and preventable cause of disability and mortality in the United States [1]. Despite wide-spread motivation to quit smoking among smokers [2] and efficacious smoking cessation treatments [3], many people who successfully quit smoking relapse [4]. The omnipresense of smoking-related cues (e.g. ashtrays, other smokers) in their environment increases the urge to smoke, making relapse more likely [5]. As such, there is an ongoing need for the development of novel and effective smoking cessation treatments or treatment adjuvants that reduce cue-provoked urges. Cue exposure therapy (CET) targets cue-provoked urges by repeated exposure to drug-related cues (e.g. cigarettes) without the actual reinforcing effects of drug use (e.g. nicotine). That is, CET extinguishes the cue-provoked urge to use the drug [6–9]. However, its efficacy is often short-lived due to limited generalization of extinction learning to other contexts [10], such as the many locations and situations where drug use occurs. This is known as the renewal effect [11,12]. Augmented reality (AR) has recently emerged as a rapidly developing technology that has substantial potential [13,14] for treating addiction, including tobacco use [15]. AR involves the real-time insertion of digitally-created images into a user’s natural environment as viewed through a screen or headset. In recent years it has been utilized for entertainment (e.g. Pokémon Go), retail sales (e.g. viewing potential household products as they might appear in one’s home), and medical treatment and training [16]. AR contrasts with virtual reality (VR), in which a user is immersed as much as possible into a fully digital environment as experienced via a headset and other sensory apparati. Although VR has been used to treat addiction [17], AR has functionality that addresses limitations of VR (e.g. high cost in development, low realism, not the user’s actual environment [15]). The ability of AR to insert digitally created objects into the user’s environment in real-time within the user’s natural environment directly addresses limitations in the generalizability of extinction learning. The AR object can be either still or moving and placed in virtually an infinite number of environments (e.g. home, bars, social situations), which is likely to facilitate exposure to drug cues in typical drug use settings while also enhancing the immersive experience and ecological validty.

AR also has several unique practical advantages, as compared to VR, including relatively low cost in developing digital objects, high degree of realism given the simplicity of the AR objects, and real-time exposure by inserting AR objects into the user’s natural environment [15] (e.g. an ashtray on the user’s end table in their living room). AR can be implemented via most personal mobile device, including smartphones without extra equipment such as headsets or helmets as in VR. Thus, individuals using AR for substance use treatment can view the AR objects superimposed on their typical real-world drug using environment. Given the rapid increase in smartphone use, even among low-income populations (e.g. 76% of low-income populations own a smartphone in the U.S. [18]), AR has great clinical and therapeutic potential in accessibility and affordability. AR has been applied to CET for phobias and has demonstrated preliminary [13,14,19] and comparable efficacy in reducing fear and anxiety in small animal phobias when compared to in-vivo exposure [19]. However, the applicability of AR for the treatment of addiction has not yet been tested.

**Figure 1.**
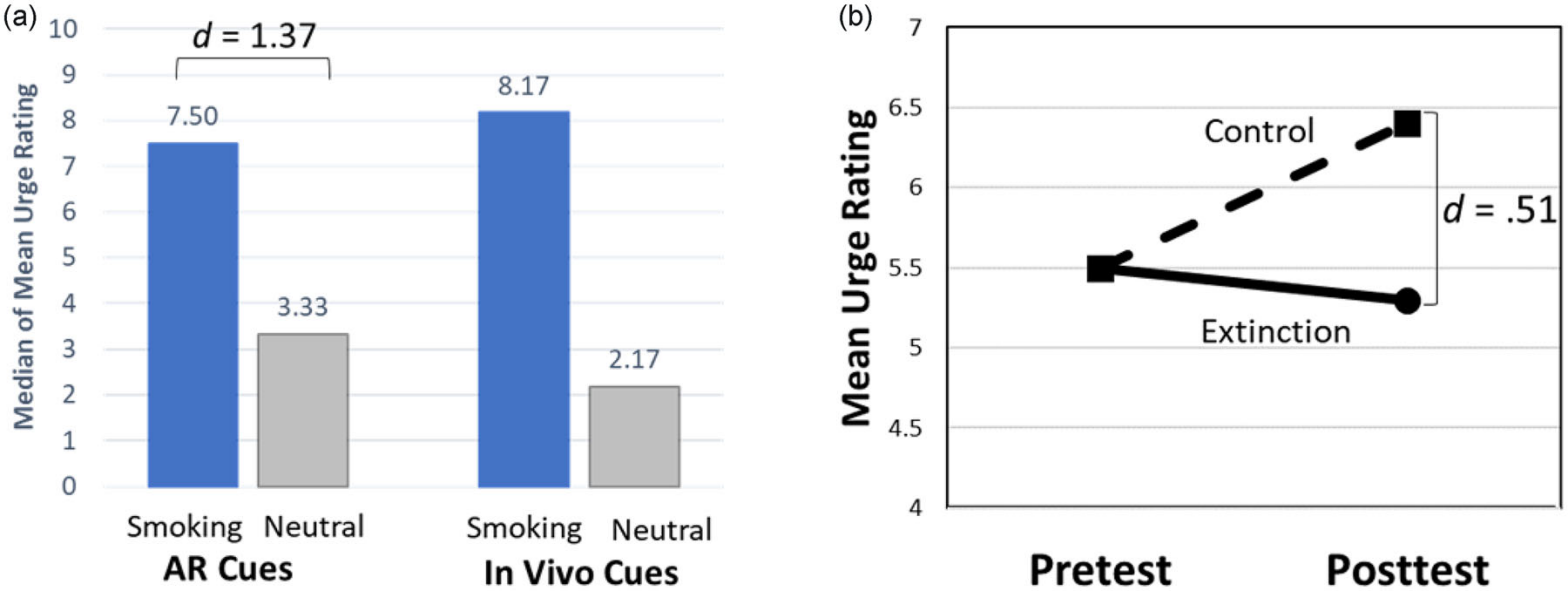
Summary of previous findings. (a). Median of each participant’s mean urge ratings in response to smoking and neutral cues presented via AR and in vivo (adapted from Brandon et al. 2020). (b) Mean urge ratings at pretest and posttest of AR cigarette cues for Extinction vs Control conditions, demonstrating extinction at posttest (adapted from Yang et al. 2022). AR: Augmented Reality.

Our team developed a basic AR smartphone app for the delivery of CET as an adjuvant strategy for smoking cessation treatments, and we evaluated whether it met the two necessary conditions for CET: (1) that the smoking cues elicited cue-reactivity, particularly self-reported urge to smoke and (2) that repeated exposure to the cues produced evidence of extinction. Both of these conditions were met in a controlled laboratory-based setting among daily smokers [20–22]. Specifically, we developed 6 smoking paraphernalia AR objects (e.g. cigarette, ashtray) activated via a smartphone AR app. Our earlier studies demonstrated that the AR cues were perceived as realistic and well-integrated into the user’s environment [20,21]. Additionally, AR smoking cues demonstrated higher cue reactivity with a large effect size as compared to AR neutral cues, with cue-reactivity magnitude comparable to in vivo smoking cues (Figure 1(a), [20]). In a subsequent proof of concept study, daily smokers in acute abstinence were randomized to either extinction (viewed AR smoking cues) or control (viewed AR neutral cues) conditions in which they completed a single session of multiple trials of cue exposure using the AR app in a laboratory setting [22]. We found that posttest cue-provoked urge was lower in the extinction condition than in the control condition with a moderate effect size, suggesting that cue reactivity to AR smoking cues can be extinguished (Figure 1(b), [22]). Together, these studies provide foundational evidence for the potential clinical utility of AR cues in CET for addiction with a focus on smoking behavior.

To advance the AR app toward evaluation in a full-scale randomized controlled trial (RCT), the current study assessed its usability and acceptability among daily smokers not currently making a quit attempt in their typical natural environments. Although the study was not powered to test clinical outcomes, we describe proxies of behavior change (e.g. motivation to quit, cessation self-efficacy, urges to smoke) and changes in cigarettes per day.

## Method

### Participants

Daily smokers were recruited between November 2021 and February 2022 through online advertisements and a participant database at the Tobacco Research and Intervention Program at Moffitt Cancer Center. The final sample size was determined based on the recommended sample size for usability studies being at least *N* = 20 to capture 98.4% of potential usability problems [23]. Inclusion criteria were: (1) ≥ 18 years of age, (2) currently smoking ≥ 3 cigarettes per day (CPD) for the past year, (3) motivated to quit smoking within the next month (via a single question reflecting the ‘preparation’ stage of the Transtheoretical Model, [24]), (4) having an AR-compatible smartphone that the participant was willing to use during the study, (5) having a valid home address and phone number, and (6) being able to speak, read, and write in English. Exclusion criteria were the regular use of other tobacco products (>30% of the time) and having a household member already enrolled in the study. Participants were aware that they would be asked to test and provide feedback on an app under development, and that they would not be receiving smoking cessation assistance per se.

### Procedure

The study was a single-arm pilot trial that tested the usability and acceptability [25,26] of an AR app for smoking cessation for 7 days among daily smokers. Given this was a usability study rather than a clinical trial, one week seemed sufficient to obtain feedback about the functionality of the AR app. All study procedures were conducted remotely. Participants were phone screened to determine eligibility. Eligible participants provided verbal consent to participate in the study and were aware that their data would be fully anonymized in the publication of the findings. Participants were then provided instructions on the AR app and sent an electronic link to a baseline survey. Upon completion of the baseline survey via REDCap, materials comprising participant procedures, a manual on how to download and use the AR app (iOS or Android version), the National Cancer Institute’s *Clearing The Air* smoking cessation booklet, and the contact information for the Florida state tobacco Quitline were mailed to each participant. Study staff then re-contacted participants to ensure that they downloaded the AR app and to answer any questions.

Participants were instructed to use the AR app at least 5 times per day over the next 7 days in locations and situations where they typically smoked (e.g. home, outdoors) and when they experienced an urge to smoke. We selected 5 daily uses as the target for this study in an initial attempt to balance the need for adequate extinction duration across multiple contexts with the desire for reasonable participant burden for encouraging adherence. At the start of each day, participants were asked to indicate in the app how many cigarettes they smoked the day before. One day after the completion of the 7-day AR app use, a link to complete a follow-up survey via REDCap was sent to participants, after which study staff contacted participants to conduct a semi-structured phone interview to obtain additional feedback on the AR app (not reported in this paper). Participants were compensated up to $80. The project was registered in clinicaltrials.gov on September 24, 2019 (NCT04101422) and addressed the ‘Testing AR Application’ aim, using an independent sample. All procedures were approved by the Advarra Institutional Review Board (Protocol Number 20007). Data and study materials will be available upon reasonable request.

### Augmented reality app and cue exposure session

The AR app used in our previous lab-based studies was developed on the Unity^®^ platform and ran on Apple 10xr iPhones provided to participants. The app included six smoking and six neutral AR images [20–22]. Data collected by the app were transferred to the study desktop computer following each session. The initial app presented each AR cue for 1 minute in a given cue exposure session with a fixed number of AR cues presented. For the present study, the AR app was updated for field testing as follows: (1) one additional AR cue, coffee, resulting in 7 AR cues in total; (2) inclusion of a question on the number of cigarettes smoked the prior day; (3) real-time data transmission from the app to the institutional server; (4) added Android compatibility; and (5) variable presentation length of each AR trial (30 to 60 seconds) and number of AR cues per session (3 to 7 cues). The range of trial duration was selected to provide adequate exposure without threatening engagement and compliance due to participant boredom or frustration. Participants received a daily reminder to complete the daily assessment at 10:00am. Upon completion of the daily assessment, participants were prompted to conduct an AR cue exposure session at that time or select to delay it. If they opted to delay the AR session, the app displayed, ‘Okay, no problem. Remember to come back and do the AR session at least 5 times a day.’ Furthermore, after completing an AR session, the app displayed, ‘Well done! You finished an AR session. Please complete 5 each day!’ There was no further reminder notification to complete a session. Whenever a participant opened the AR app, the home screen appeared, and participants could select to complete an AR cue exposure session. Menus for a practice session (using an AR rubber duck cue) and contact information of the study team were also available from the home screen.

**Figure 2.**
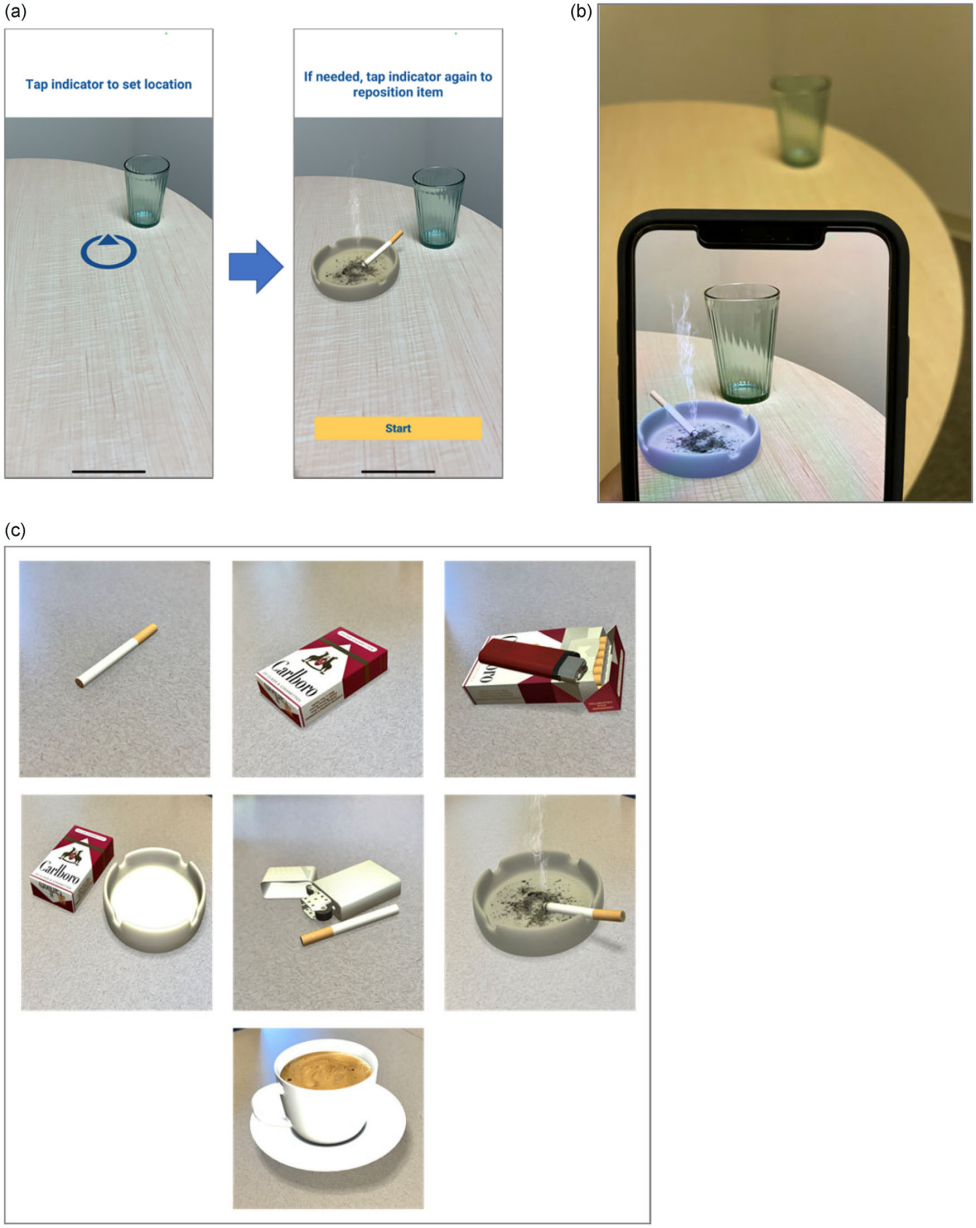
Overview of the AR app. (a) Placement of AR cue within the AR app. (b) Sample AR trial from a participant’s view. (c) Seven AR cues within the AR app. AR: Augmented Reality.

In each cue exposure session, participants were instructed to move the phone to aim the camera at a flat surface where they would typically place smoking-related paraphernalia until a blue placement circle appeared on a flat surface (Figure 2(a)). The circle would appear once the app identified a horizontal plane (flat surface). The participant could then move the circle to any location on that plane. Once the circle was positioned in the desired location, participants tapped the circle to trigger the AR cue to appear at that location. They then pressed the ‘Start’ button to begin the cue exposure session (Figure 2(b)) and viewed the AR cues by viewing the app on their smartphone. Each session consisted of 3 to 7 trials of cue exposure. The order, presentation length, and number of AR cues were randomized. To enhance the immersive experience with the AR cues, participants were instructed to approach the AR cue from different angles and distances. The AR cues consisted of six smoking-related proximal cues (i.e. cigarette, pack of cigarettes, pack and lighter, pack and ashtray, cigarette and lighter, and a lit cigarette in an ashtray with smoke motion) and one smoking-related distal cue (i.e. a cup of coffee; Figure 2(c)). Each cue was presented for 30 to 60 seconds. At the beginning of each session and following each cue exposure trial, participants reported their current urge level by answering a 1-item question on the app.

### Measures

#### Baseline survey

Self-reported demographic information (age, ethnicity, race, biological sex, marital status, education, and income), tobacco use history (average CPD on the days they smoked in the past month), and overall strength of urge to smoke in the past month (three response options: none/slight, moderate/strong, and very/extremely strong) were assessed. Nicotine dependence was assessed by the 6-item Fagerström Test for Cigarette Dependence (FTCD; [27,28]). The FTCD is a standard self-report measure that assesses nicotine dependence that uses a mix of yes/no items and multiple-choice items. Its total score ranges from 0 to 10 (higher = greater dependence). To describe behavior change in smoking-related variables, three well-established standardized self-report measures that have demonstrated adequate psychometric properties in previous studies were administered: (1) The Contemplation Ladder [29] to assess motivation to change smoking behavior, (2) 10-item Short-form Abstinence-Related Motivational Engagement Scale (ARME; [30]) to assess ongoing engagement in abstinence, and (3) 9-item Self-Efficacy Scale – Smoking [31] to measure perceived confidence for not smoking in situations where they typically smoke (e.g. social situations, when experiencing negative affect). The Contemplation Ladder asks an individual to choose one motivation level out of 10 levels (0 = No thought of quitting smoking cigarettes, 10 = Taking action to quit smoking cigarettes [29]). The ARME uses 7-point Likert scale (1 = Completely Disagree, 7 = Completely Agree) and the total score ranges from 7 to 70 (higher = greater ongoing engagement in abstinence [30]). Lastly, the Self-Efficacy Scale is rated on 5-point Likert scale (1 = Not at all confident, 5 = Extremely confident) and its total score ranges from 9 to 45.

#### Augmented reality app measures

Within the AR app, participants completed a single-item assessment of daily cigarette smoking (i.e. ‘How many cigarettes did you smoke yesterday?’). Engagement with the app (i.e. number of sessions completed per day, number of discrete days the app was used, number of AR sessions completed on day the app was used, and number of AR cues viewed on days the app was used) was also captured.

#### Follow-up survey

Usability and acceptability were measured using the following self-report questionnaires at follow-up based on the literature on mHealth usability [25,26]: (1) a 3-item reality/co-existence questionnaire that was developed by the team to assess reality (‘How real did the object seem to you?’), environment co-existence (‘How well did the object appear to be part of the scene?’), and user co-existence (‘How much did you feel the object was right there in front of you?’) on a 10-point Likert scale (1 = Not at all, 10 = Very real/very well/very much), (2) the 10-item System Usability Scale (SUS) that has been validated in prior studies [32] to assess learnability and usability of the system (e.g. ‘I thought the app was easy to use’) on a 5-point Likert scale (1 = Strongly Disagree, 5 = Strongly Agree; higher score = greater usability; [33]), (3) a two-item scale to assess ease of use at the beginning and at the end of the week (How easy/difficult was it to use the app?) on a 5-point Likert scale (1 = Very difficult, 5 = Very easy), (4) a single item to assess ease of learning (How many days did it take to get comfortable using the app?; 7 response options: 1 to 7 days), (5) two items to assess satisfaction (Would you recommend this app to a friend or family member to help them quit smoking?; Overall, how satisfied were you with the app?) on a 4-point Likert scale (1 = No/Quite dissatisfied, 4 = Yes/Very satisfied), and (6) a single-item to assess usefulness (Would this app appeal to you if you were currently attempting to quit smoking? yes/no). Besides the SUS, the rest of the usability and acceptability measures in the follow-up survey reported in the current study were developed by our study team and mimic those used in previous research in this area [20,21].

To describe change in motivation and self-efficacy at posttest, the Contemplation Ladder, ARME, and Self-Efficacy Scale were administered. To describe changes in the strength of urge to smoke, a single-item measure of smoking urge over the past week was collected (3 response options: non/slight, moderate/strong, and very/extremely strong). Although participants were not offered smoking cessation treatment and not actively attempting to quit or reduce smoking, past week smoking behavior (average CPD on the days they smoked) was assessed. Participants were also asked where (sample options: home, outdoors, work, bars), when (sample options: with food, while doing an activity, first thing in the morning, when feeling a negative emotion), and with whom (sample options: with familiar people, with other smokers, alone) they used the AR app. Additionally, the number of participants who contacted the study team for additional help using the app was tracked.

### Data analysis

Descriptive analyses (e.g. means, SDs, proportions) were conducted on the study variables including demographics, self-report measures on usability and acceptability, app use, motivation, self-efficacy, and change in CPD. Although the current sample was not powered for inferential statistics, paired t-tests and McNemar’s test were conducted to explore changes in the proxy variables between baseline and follow-up.

## Results

### Sample characteristics

**Figure 3.**
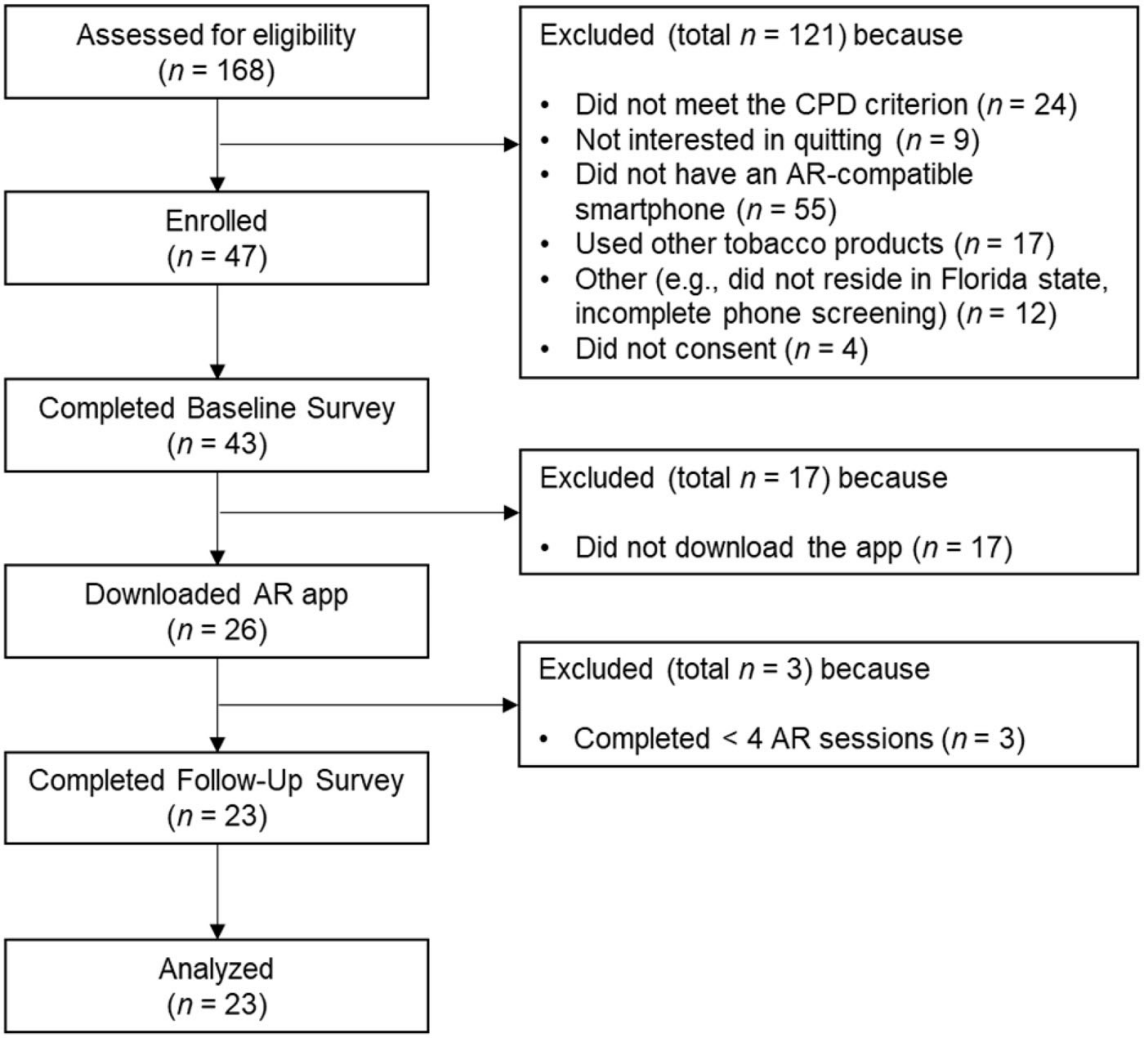
CONSORT diagram. CPD: Ciagarettes Per Day; AR: Augemented reality.

Figure 3 presents the CONSORT diagram. We enrolled 43 participants who returned the baseline survey. Of these, 26 participants downloaded the app onto their smartphones. Of the 17 who did not download the app, we learned that at least 7 determined that their phones were not AR compatible. Three of the participants who downloaded the app did not complete the AR sessions at least 4 times, leaving 23 participants for analysis. We determined that completing at least 4 AR sessions would provide the minimum AR app experience necessary to provide feedback on the app. Table 1 presents baseline data, including demographics (78.3% female; 13.0% Hispanic/Latinx; 17.4% Black/African American), smoking characteristics, previous experience with AR apps, and initial interest in using the AR app. Participants were moderately nicotine dependent and reported a strong interest in using the AR app. Approximately half reported previous experience with AR apps.

**Table 1.** Sample Characteristics at Baseline. (*N* = 23).

Variable	Mean (SD) or n (%)
Age	43.43 (11.07)
Biological Sex: Female	18 (78.26%)
Sexual Orientation: Lesbian, Gay, Bisexual	2 (8.70%)
Ethnicity: Hispanic/Latinx	3 (13.04%)
Race	
White	17 (73.91%)
Black/African American	4 (17.39%)
Other	2 (8.70%)
Marital Status	
Single	14 (60.87%)
Married/Living with significant other	3 (13.04%)
Other	6 (26.09%)
Education	
GED	3 (13.04%)
High school diploma	2 (8.70%)
Some college	9 (39.13%)
Tech School/Associate/Undergraduate degree	8 (34.78%)
Some graduate school	1 (4.35%)
Annual Household Income: < $30,000	11 (47.83%)
Smoking Characteristics	
Cigarettes Per Day in past month	14.91 (6.57)
FTCD	5.00 (2.02)
Plan to quit smoking within 30 days (Yes)	21 (91.30%)
Another member in household is a smoker (Yes)	8 (34.78%)
Previous Experience with AR	
Ever used	
No	9 (39.13%)
Yes	11 (47.83%)
Don’t know	3 (13.04%)
Frequency of AR app use	
Daily	2 (8.70%)
1-6 days/week	3 (13.05%)
Less than weekly	8 (34.78%)
Never/Don’t know	10 (43.48%)
Interest in Using AR App (10-point scale)	9.48 (1.04)

FTCD: Fagerström Test for Cigarette Dependence.

### Usability and acceptability

Table 2 presents the descriptive statistics of the usability and acceptability outcomes. Seventeen participants (74%) used the app at least 5 days and 12 (52%) used the app daily for 7 days. The mean number of AR sessions completed on days the app was used was 3.8 (SD = 1.5) and the mean number of AR cues viewed was 22.9 (SD = 8.6) on days the app was used. Participants reported that the top three places where they used the AR app were home (91%), car (48%), and outdoors (44%). The top three occasions when they reported using the app included with coffee (52%), before going to bed (52%), and when bored (52%). Participants also reported using the app while doing an activity (48%) or first thing in morning (48%). Lastly, participants primarily used the app when they were alone (83%) followed by being with familiar people (35%) and/or other smokers (22%).

**Table 2.** Usability and acceptability measures (*N* = 23).

Usability & Acceptability Variable	M (SD) or n (%)
Frequency of App Use	
Number of days^a^ the app was used	5.78 (1.62)
Number of sessions on days the app was used	3.81 (1.46)
Number of AR cues viewed on days the app was used	22.89 (8.58)
Reality/Co-Existence of AR Cues (10-point scale)	
Realism	7.91 (1.88)
Environmental Coexistence	7.96 (2.31)
User Coexistence	8.04 (2.12)
System Usability Scale (0-100 scale)	79.46 (17.14)
Ease of Using App (5-point scale)	
Perceived ease at beginning	3.83 (1.40)
Perceived ease at end	4.13 (1.32)
Overall perceived ease at follow-up survey (Agree)	20 (86.95%)
Ease of Learning the App	
Number of days for learning to use the app	1.91 (1.59)
Contacted staff for additional help (Yes)	4 (17.39%)
Satisfaction	
Would recommend the app (Yes)	15 (65.22%)
Overall satisfaction with the app (Mostly/Very)	16 (69.57%)
Appeal of the App for Quitting (Yes)	11 (47.82%)

^a^Indicates the total number of discrete days the app was used.

On the follow-up survey, participants reported an overall positive experience with the AR app. They reported that the AR cues were highly realistic, were well-integrated in the environment, and appeared as if the cues were right in front of them. The AR app was perceived as easy to use and learn as indexed by the System Usability Scale (*M* = 79.5, SD = 17.1). There was a statistically nonsignificant increase in the perceived ease of using the app over time (*t*(22)=-1.37, *p*=.184) and 87% reported that the app was overall easy to use at the follow-up survey. The average number of days to become comfortable with the app was 2 with the majority reporting only 1 day (*n* = 15; 65.2%), and only 17% (*n* = 4) asked for additional help from the study team. The 4 participants who asked for additional help provided the following reasons: trouble downloading because they had the wrong app name; incorrect ID number; trouble placing the indicator; and asking where the practice tutorial was located. High satisfaction was reported such that 65% reported that they would recommend the app to a friend or family member to help them quit smoking and 70% expressed overall satisfaction with the app. Lastly, 48% reported that the AR app would be appealing to them if they were currently attempting to quit smoking.

### Smoking behavior

Table 3 presents the descriptive statististics of the smoking variables at baseline and follow-up. No significant changes were found on the Contemplation Ladder, the ARME, or the Self-Efficacy Scale. However, both the proportion of participants who reported strong urge to smoke and CPD significantly declined. Specifically, participants were less likely to experience very/extremely strong urges at follow-up (17%) than at baseline (57%, *p*<.01). Participants also reported smoking slightly fewer cigarettes at follow-up as compared to baseline (*t*(22)=-2.90, *p*<.01). In particular, 13 participants (57%) reported having reduced the number of cigarettes smoked in the past week at follow-up, with an average reduction of 26% among them (*M* = 3.7, SD = 1.8).

**Table 3.** Smoking-related variables at baseline and follow-up survey (*N* = 23).

Variable	Baseline	Follow-Up
Motivation to Quit (M, SD)		
The Contemplation Ladder^a^	7.40 (1.76)	6.19 (2.29)
ARME	50.39 (9.69)	51.96 (8.35)
Self-Efficacy Scale – Smoking (M, SD)	22.04 (4.19)	22.87 (7.12)
Strength of Urge to Smoke^b^ (*n*, %)**		
None/Slight	1 (4.35%)	1 (4.35%)
Moderate/Strong	9 (39.13%)	18 (78.26%)
Very/Extremely Strong	13 (56.52%)	4 (17.39%)
Cigarettes Per Day in past week (M, SD)**	14.91 (6.57)	13.17 (6.63)

** *p*<.01.

^a^Data from 15 participants at baseline and from 16 participants at follow-up.

^b^Past month at baseline and past week at follow-up.

ARME = 10-item Abstinence-Related Motivational Engagement Scale.

## Discussion

Previous laboratory-based research established that the AR app met the necessary mechanistic criteria underlying CET—cue reactivity and extinction. The current study extended that research by examining the usability and acceptability of the AR app among daily smokers in their natural environments.

Usability was supported by high ratings across several measures. Consistent with our earlier findings in laboratory settings [20,21], AR cues were perceived as highly realistic and well-integrated in the user’s natural environment. Perceived reality/co-existence is one crucial component for an immersive AR experience [34], which was met in our study. Although only 52% of participants used the AR app every day for a full week, the majority used it on at least 5 days, supporting the feasibility of the AR app. Regarding ease of use, the average rating on the System Usability Scale was very high, and participants reported increased ease of use over time. Similarly, participants reported high learnability, with the majority reporting feeling comfortable using the app within the first day. Anecdotally, difficulties appeared to be related to particular viewing conditions (e.g. dim light) and idiosyncratic limitations with some Android phones. Moreover, few participants reached out for additional help from the study team.

Acceptability of the app was deemed moderate to high. Overall, participants reported high satisfaction with the app, although only half of participants reported that the app would be appealing if they were currently attempting to quit. However, this is not surprising given that the current study focused on the usability of the extinction trials, so the basic app did not include other user engagement features that will be added later, nor did we provide other standard components of smoking cessation interventions. Whereas app functionality regarding CET was our top priority at this developmental phase, other features such as gamification or progress feedback were not yet implemented, nor was it yet integrated into a smoking cessation program. Thus, it is possible that our acceptability ratings were impacted by the somewhat bare-bones nature of this initial version of the app.

Exploratory analyses revealed some small but significant changes in smoking proxies and behavior over the week of app use. No changes were found in the cessation motivation or self-efficacy indices over this short time-frame. However, both strong urge to smoke and CPD decreased from baseline to follow-up. These are the clinical indices that theoretically should be most affected by CET, so it is encouraging that we detected a change signal even within this feasibility trial.

It is important to acknowledge the limitations of this study, particularly in comparison to a full-powered clinical trial. The sample size was underpowered for inferential statistics across participants, participants were not enrolling in a smoking cessation program per se, the CET intervention lasted only a week, and this version of the AR app included only AR presentation without features to enhance engagement (e.g. gamification, reminders to complete the AR sessions) or additional smoking cessation assistance. Moreover, this single-armed trial did not include control or comparison arms, which limits causal conclusions about the exploratory longitudinal findings. Despite these limitations of a usability/acceptability trial, the study yielded encouraging findings with respect to both the usability and acceptability of an AR app that presents smoking-related stimuli in participants’ natural settings. One remaining concern is the availability of AR-compatible smartphones, given that a relatively high proportion of potential participants were excluded because their devices were not AR-compatible. It is likely, though, that these individuals were using older devices, since most contemporary smartphones now have this capability. Thus, this concern should attenuate with the diffusion of newer technology.

Given the novelty of clinical AR apps, several research questions remain. First, this app was envisioned to be integrated into a comprehensive smoking cessation intervention. Therefore, research is needed to determine how best to pair the app with an existing treatment. Relatedly, the incremental efficacy of the AR app for smoking cessation should be tested in comparison to standard smoking cessation interventions (e.g. cognitive behavioral therapy, pharmacotherapy). There are also various theoretical, functional and implementation questions that have been elaborated elsewhere [15]. Other sensory modalities such as odor and sound could also be explored to enhance the realism and engagement of the AR stimuli [13]. Collecting daily information on the place, time, and social situation during the extinction trials would contribute to a better understanding of how the app was used.

Finally, the current results, together with our previous laboratory findings, have demonstrated that a smoking-related AR app elicits cue reactivity [20,21], produces extinction [22], and is feasible and acceptable to participants. Thus, not only does this suggest that CET for smoking cessation may be an effective and feasible treatment option, but AR-based CET could also be considered for the treatment of addictive behaviors involving other substances, such as alcohol or illicit drugs. AR-based CET offers the advantage of conducting cue-exposure in users’ diverse substance-use environments—which should enhance the generalizability and maintenance of extinction—without the risks associated with the presence of actual substances or their paraphernalia. Thus, AR offers the potential to break through the barrier of the renewal effect, which has limited the utility of CET for smoking and other substance use [12]. Further, aside from extinction-based treatment, AR could also be utilized to train coping responses such as cognitive-behavioral or mindfulness skills that can be used when individuals encounter naturally-occurring high-risk situations [15]. Moreover, once AR headsets or glasses become more available and affordable, users will be able to view AR images directly, rather than via a smartphone screen, which should further improve realism, engagement, and, ultimately, efficacy. Limitations of AR for CET should also be acknowledged. These include reliance on a single sensory modality (vision) to date, in comparison to in vivo stimuli that may include tactile, olfactory, and auditory cues. Additionally, some drug-related stimuli, such as the presence of particular individuals, are currently too complex to be created as AR images. Finally, the lack of direct therapist involvement during the CET sessions, while offering flexibility, may come at a cost of extinction session control, consistency, engagement, and adherence. Future research should examine the cost-benefit ratio of CET with respect to its advantages and limitations.

The current study was the first, to our knowledge, to test and demonstrate both usability and acceptability of an AR app for smoking cessation in the real world. Together with our previous laboratory studies, this research provides evidence supporting the clinical potential of AR-based CET for smoking cessation and relapse prevention. The current findings set the stage for an RCT testing the AR app as an adjuvant therapy for treating tobacco dependence, with potential applicability to other substances.

## Author contributions

**Min-Jeong Yang:** Writing – original draft, Formal analysis, Interpretation of the data, Visualization, Revising it critically for intellectual content, Final approval of the version to be published

**Karen O. Brandon:** Project administration, Data curation, Formal analysis, Interpretation of the data, Visualization, Writing – review & editing, Revising it critically for intellectual content, Final approval of the version to be published

**Steven K. Sutton:** Data curation, Formal analysis, Interpretation of the data, Visualization, Writing – review & editing, Revising it critically for intellectual content, Final approval of the version to be published

**Marloes Kleinjan:** Study Design, Interpretation of the data, Writing – review & editing, Revising it critically for intellectual content, Final approval of the version to be published

**Leslie E. Sawyer:** Study Design, Interpretation of the data, Writing – review & editing, Revising it critically for intellectual content, Final approval of the version to be published

**Thomas H. Brandon:** Conceptualization, Study Design, Funding acquisition, Investigation, Methodology, Interpretation of the data, Writing – review & editing, Revising it critically for intellectual content, Final approval of the version to be published

**Christine Vinci:** Conceptualization, Study Design, Funding acquisition, Investigation, Methodology, Interpretation of the data, Writing – review & editing, Revising it critically for intellectual content, Final approval of the version to be published

All authors approved the final version of the manuscript and agreed to be accountable for all aspects of the work.

## Data Availability

The data that support the findings of this study are available from the corresponding author, CV, upon reasonable request.

## Clinical trial registration details

The current study design is registered in clinicaltrials.gov (NCT04101422); see https://clinicaltrials.gov/ct2/show/NCT04101422.
